# NOBODY CAN DRAG ME DOWN: AGING AS A DRAG PERFORMER

**DOI:** 10.1093/geroni/igad104.1397

**Published:** 2023-12-21

**Authors:** Laura Donorfio, Kathleen Henneberry, Brian Chapman

**Affiliations:** University of Connecticut, Waterbury, Connecticut, United States; University of Connecticut, Waterbury, Connecticut, United States; University of Connecticut, Waterbury, Connecticut, United States

## Abstract

This presentation will detail the findings of an exploratory, qualitative research study examining the intersection of aging and drag performance. Even though drag queens have achieved significant levels of visibility in mainstream entertainment and have become increasingly present topics of discussion in news outlets, modern media, and even political landscapes, they are an understudied and underrepresented population (Knutson et al., 2020; O’Brien, 2018). Older drag queens have yet to be studied in the social science literature (Henneberry et al., 2022). “Drag Queens possess unique characteristics as a subpopulation of the lesbian, gay, bisexual, and transgender (LGBT) community that warrant exploration” (Knutson, Koch, Sneed, & Lee, 2018). This research aims to understand and represent the relatively understudied population of drag queens in academic literature while drawing attention to the specific sub-population of older adults in the profession. A semi-structured interview protocol and thematic analysis was utilized to understand the unique perspectives of self identified drag performers over the age of 50 (n=15; ages 54-94). Examining drag artistry and expression through the lens of aging has revealed themes of drag as a form of armor, ageism within the community, and shifts in social perceptions of drag performers. This presentation will introduce the term “dragism” in an attempt to explain the discrimination against drag queens within the LGBTQ+ community, as well as the interplay of both dragism and ageism for the participants of this study (Donorfio et al., 2022).

